# A Gene in the Process of Endosymbiotic Transfer

**DOI:** 10.1371/journal.pone.0013234

**Published:** 2010-10-06

**Authors:** Kateřina Jiroutová, Luděk Kořený, Chris Bowler, Miroslav Oborník

**Affiliations:** 1 Institute of Parasitology, Biology Centre, Academy of Sciences of the Czech Republic and Faculty of Science, University of South Bohemia, České Budějovice, Czech Republic; 2 Institut de Biologie de l'Ecole Normale Supérieure (IBENS), CNRS UMR8197, Ecole Normale Supérieure, Paris, France; Montreal Botanical Garden, Canada

## Abstract

**Background:**

The endosymbiotic birth of organelles is accompanied by massive transfer of endosymbiont genes to the eukaryotic host nucleus. In the centric diatom *Thalassiosira pseudonana* the Psb28 protein is encoded in the plastid genome while a second version is nuclear-encoded and possesses a bipartite N-terminal presequence necessary to target the protein into the diatom complex plastid. Thus it can represent a gene captured during endosymbiotic gene transfer.

**Methodology/Principal Findings:**

To specify the origin of nuclear- and plastid-encoded Psb28 in *T. pseudonana* we have performed extensive phylogenetic analyses of both mentioned genes. We have also experimentally tested the intracellular location of the nuclear-encoded Psb28 protein (nuPsb28) through transformation of the diatom *Phaeodactylum tricornutum* with the gene in question fused to EYFP.

**Conclusions/Significance:**

We show here that both versions of the *psb28* gene in *T. pseudonana* are transcribed. We also provide experimental evidence for successful targeting of the nuPsb28 fused with EYFP to the diatom complex plastid. Extensive phylogenetic analyses demonstrate that nucleotide composition of the analyzed genes deeply influences the tree topology and that appropriate methods designed to deal with a compositional bias of the sequences and the long branch attraction artefact (LBA) need to be used to overcome this obstacle. We propose that nuclear *psb28* in *T. pseudonana* is a duplicate of a plastid localized version, and that it has been transferred from its endosymbiont.

## Introduction

Endosymbiotic events that have led to the evolution of plastids have been accompanied by fundamental genetic processes. Massive transfer of endosymbiont (plastid) genes to the host nucleus represents the most remarkable phenomenon [1], [2]. Eukaryotic nuclei thus contain not only original eukaryotic genes but also various genes transferred to it during endosymbiotic and lateral gene transfers. The estimated number of plastid genes transferred to the host nucleus during primary endosymbiosis is around 2000 [2]. Such organellar genes that became localized in the nucleus are translated in the cytosol and the proteins are targeted to the appropriate organelle [3]. Consecutive events such as secondary or tertiary endosymbioses mix the host genome even more extensively. Secondary endosymbiosis is the process in which a eukaryotic heterotroph engulfed a primary plastid-containing eukaryotic alga which subsequently evolved to become a complex plastid surrounded by three or more membranes. During tertiary endosymbiosis the engulfed alga is thought to already contain a secondary plastid [1], [4].

**Figure 1:**
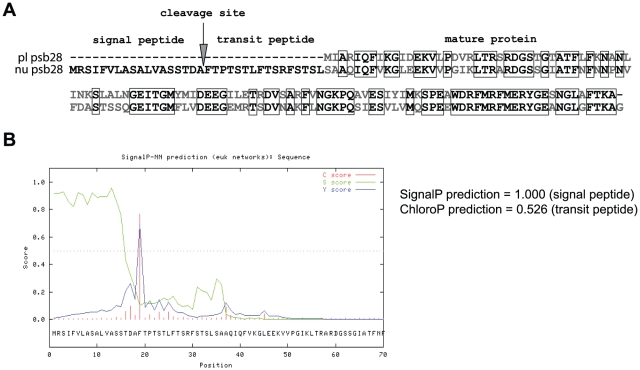
Plastid- and nuclear-encoded Psb28 proteins in *T. pseudonana*. **A.** Amino acid alignment of nuclear-encoded Psb28 protein (nuPsb28) from the genomic sequence of *T. pseudonana* with the plastid-encoded homologue (plPsb28). Identical amino acid residues are framed in black, other residues are printed in grey. **B.** Prediction of targeting sequences in the nuPsb28 using SignalP (signal peptide) and ChloroP (chloroplast transit peptide).

Before diatom genomes were sequenced, no convincing evidence for direct transfer of genes from secondary plastid to the secondary host nucleus was available. When the first centric diatom genome was annotated, promising candidate genes for having been transferred directly from the plastid to the secondary host nucleus were identified [5]. A rather unexpected finding concerned Psb28, that in the centric diatom *Thalassiosira pseudonana* was found to be encoded in both the nuclear and plastid genomes. Conversely, the nuclear-encoded version is putatively targeted, according to computer predictions, to the diatom complex plastid (see Figure 1 for details). This indicates that *psb28* could represent a gene undergoing transfer to the diatom nucleus during the process of endosymbiotic gene transfer in *T. pseudonana*. As mentioned above, originally organellar-, now nuclear- encoded proteins are targeted to plastids. However, the modes of protein targeting substantially differ between primary and complex plastids. In primary hosts (plants, green algae and rhodophytes) the protein is targeted to the organelle bounded by two membranes thanks to the N-terminal chloroplast transit peptide (cTP) [3]. In the case of diatom secondary plastids, which are surrounded by four membranes, the targeting is even more complex. The diatom presequence is composed of two parts: a signal peptide which targets the protein into the endoplasmic reticulum and subsequently over the two outer membranes into the periplastidial space [6], and a conventional chloroplast transit peptide which delivers the protein into the plastid stroma over the two inner membranes corresponding to the original membranes of the primary plastid. Some nuclear-encoded proteins can be targeted only to the periplastidial space, which represents a metabolically active remnant of the algal endosymbiont cytosol.

The multiprotein membrane complex of photosystem II (PSII) contains a large number of low molecular weight proteins (Psb). Most of them are located within the cyanobacterial or plastid thylakoid membrane. The Psb28 protein is a part of this multiprotein complex. However, the identification and annotation of Psb28 has often been quite confusing. Initially, the mature 6.1 kDa PsbW protein was found in plants and chlorophytes [7]. Subsequently, the 13 kDa Psb28 photosystem II protein was incorrectly described during annotation of the *Arabidopsis thaliana* nuclear genome as a PsbW-like protein. However, both proteins differ in their molecular weight and polarity of amino acids in their sequence. The 6.1 kDa PsbW protein consists of hydrophobic amino acids that form an α-helix structure. By contrast, Psb28 (13 kDa) is a water soluble protein which is directly assembled into dimeric PSII supercomplexes. Consequently, the PsbW-like protein was later renamed Psb28 [8].

PsbW is encoded only in the nucleus of plants and green algae, although Psb28 is also found in cyanobacteria. Indeed, the gene encoding Psb28 has been found in the plastid (or cyanelle) genomes of glaucophytes [9], rhodophytes [10], cryptophytes [11] and several stramenopiles [12], [13], and recently also in the nuclei of *Emiliania huxleyi*, *Aureococcus anophagefferens, Thalassiosira pseudonana* and *Fragilariopsis cylindrus*. As already mentioned, Psb28 was shown to be both nuclear and plastid encoded in the centric diatom *T. pseudonana* and pennate *F. cylindrus*. Surprisingly, *psb28* has not been identified in the nucleus of the pennate diatom *Phaeodactylum tricornutum*, where the gene in question is found only in the plastid genome. Such discrepancy between two relatively closely related species has been explained by their surprisingly high divergence [14]. However, this suggests that endosymbiosis is a never ending ongoing process and that different taxa can substantially differ in the spectrum of genes transferred to the nucleus. Here we show evidence that both *psb28* genes are transcribed in *T. pseudonana* and that the nuclear-encoded protein is targeted to the diatom complex plastid. The extensive phylogenetic analysis demonstrates the crucial impact of nucleotide and amino acid composition of the analyzed sequences on tree topology.

## Results

We have performed an extensive search within the genomes of *T. pseudonana* [5], *P. tricornutum* [14] and *F. cylindrus* for *psb28* homologues. In agreement with previous results [5], [12], [15,] we found a single *psb28* gene in the plastid genome of the pennate diatom *P. tricornutum* and two versions of the gene in *T. pseudonana* and *F. cylindrus*, one in the nucleus, while the second is located within the plastid genome. We decided to experimentally confirm localization of the nuclear-encoded putatively plastid targeted Psb28 protein from *T. pseudonana* and to perform extensive phylogenetic analysis to specify the origin and location of the Psb28 protein in diatoms. Although a transformation system for *T. pseudonana* has been recently developed [16], we tested the localization of nuPsb28 in the pennate diatom *P. tricornutum* because transfection is more routine [17], [18].

The two versions of the gene of interest in *T. pseudonana* are quite divergent. The nuclear version contains many non-synonymous mutations and both nucleotide and amino acid sequences show surprisingly high mutual divergence. Within the nucleotide sequence, of 445 nucleotides in the plastid localized gene, 120 were substituted in the nuclear gene, which constitutes a change in 27% of the nucleotides. The nuPsb28 is 149 amino acids long and contains targeting presequences at the N-terminus necessary for targeting the protein into the complex plastid. The plastid encoded homologue of the protein consists of 114 amino acid residues. Both proteins share only 63% amino acid identity; whereas 85% can be aligned with a residue with similar biochemical properties (see Figure 1 for details).

**Figure 2:**
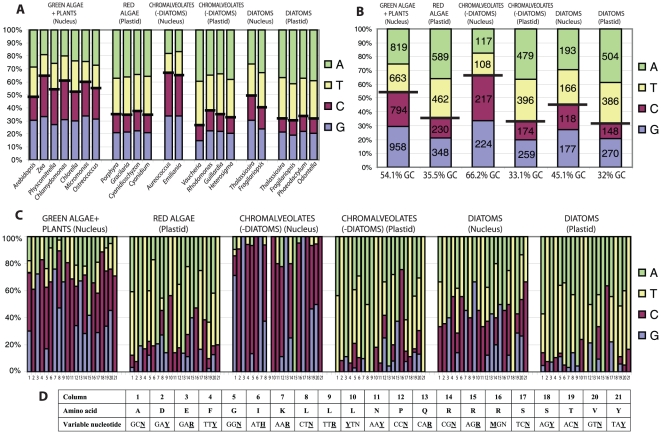
Nucleotide composition of the plastid and nuclear *psb28* genes. Figures A and B show nucleotide composition of the *psb28* gene in particular species (A) and groups (B). Figure C demonstrates the influence of the gene location on the nucleotide composition of mutated sites in synonymous mutations. The table below (D) displays particular codons and mutated sites.

When we investigated the nucleotide composition of *psb28* homologues in *T. pseudonana*, we found that it substantially differs between the plastid (67.3% A+T) and nuclear-localized genes (47.8% A+T) (see Figure 2 for details). Because the A+T content varied substantially among the *psb28* genes used for phylogenetic analysis (from 28.7% in *A. anophagefferens* to 73.0% in *Vaucheria litorea*), the nucleotide bias may influence the final topology significantly if nucleotides were used to infer trees (trees not shown).

We examined the nucleotide composition within the synonymous mutations in the *psb28* gene among all studied groups (Figure 2). It is obvious that the location of the *psb28* gene deeply influences substitutions in the mutated sites in favor of AT in the plastid localized genes and GC in the nuclear ones. Nuclear *psb28* genes from *T. pseudonana* and *F. cylindrus* seem to represent intermediate states between nuclear genes in plants, green algae and chromalveolates (other than diatoms), with the lowest AT content on one side and the plastid localized AT-rich genes on the other. This suggests that the diatom nuclear *psb28* has been transferred to the nucleus more recently when compared to the plant, green algae and the two genes from other chromalveolates. We have not detected significant amino acid bias among particular groups, because of high variability within the investigated groups. However, there can be LBA artefact due to high diversity of analyzed sequences (see Figure 2 for details).

**Figure 3:**
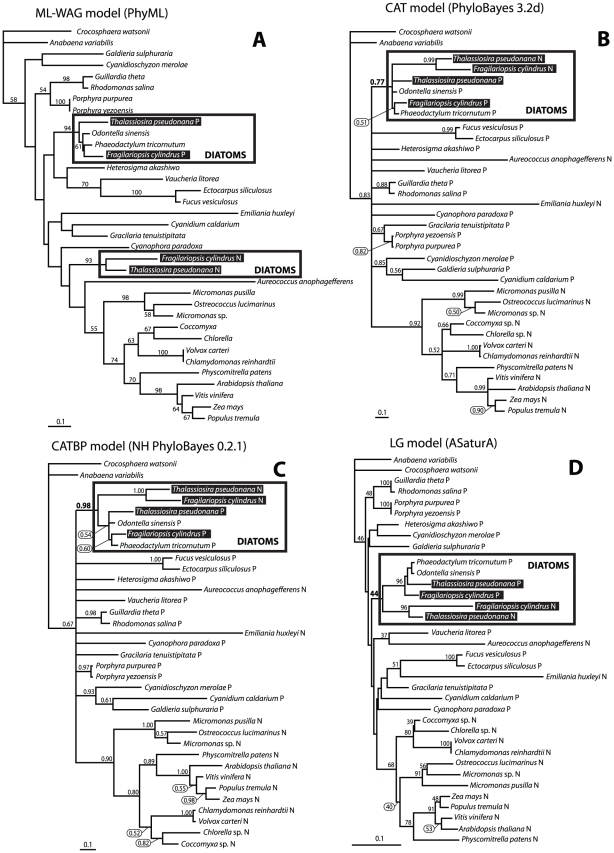
Phylogenetic trees as inferred from amino acid sequences of Psb28 (112 residues). Trees were constructed using conventional maximum likelihood (A) and methods which can deal with a compositional bias of the sequences and phylogenetic artefacts such as LBA as demonstrated here. PhyloBayes 3.2d (CAT model) (B) and NHPhyloBayes (CAT-BP model for different evolutionary rates among taxa) (C) and AsaturA (designed to deal with amino acid saturation) (D) show the expected origin of nuclear encoded Psb28 from *T. pseudonana*. It appears that *psb28* was transferred to the nucleus through endosymbiotic transfer from the diatom plastid to the diatom nucleus. Numbers above branches show Bayesian posterior probabilities (PhyloBayes 3.2d: 100000 steps, burnin = 20000, trees sampled every tenth step; HN PhyloBayes 0.2.1: 10000 steps, burnin = 2000, trees sampled each step) and bootstraps (AsaturA; 1000 replicates).

When the *psb28* genes from *T. pseudonana* were previously used to construct a phylogenetic tree, both versions of the protein clustered together, suggesting their origin from a common plastid encoded ancestor [5]. However, when we used a dataset containing more sequences that have recently become available, we obtained the expected topology only when the most sophisticated methods designed to deal with a compositional bias and long branch attraction phenomenon (LBA) (PhyloBayes 3.2d-CAT model; NH PhyloBayes 0.2.1-CATBP model; AsaturA-LG model; see Material and Methods for details) were used. All other conventional analyses (nucleotide MP, ML and amino acid based MP, ML) either placed the diatom nuclear *psb28* to the unsupported position on the root of the nuclear *psb28* genes from plants and green algae or was not able to solve the phylogenetic relationships at all (only amino acid-based ML tree is shown as an example). All the *psb28* gene sequences (*E. huxleyi*, *C. caldarium*, *C. paradoxa*, *A. anophagefferens*, *F. cylindrus* and *T. pseudonana*) that branch on the root of the nuclear clade in ML and MP analyses (Figure 3A, MP tree not shown) form quite long branches. This suggests that long branch attraction (LBA) artefact may influence ML and MP topologies.

Similarity searches within the *T. pseudonana* EST database (www.biologie.ens.fr/diatomics/EST4/index.php) revealed the occurrence of transcripts of both nuclear and plastid *psb28* versions. Surprisingly, when the plastid localized version of the *psb28* gene was searched in the non-redundant Tp EST database using BLAST [19], the best hit (C2639) was also found to encode the ribosomal protein S4, which is, however, not in the same reading frame. On the other hand, in *P. tricotnutum* the plastid *psb28* is not part of a fused transcript, and the S4 gene is not in the same region of the plastid genome. Thus the presence of *psb28* in the plastid genome of *T. pseudonana* could be the result of its fusion to the S4 ribosomal protein. In addition, we found a single EST sequence FC537861-2 corresponding to the nuclear *psb28* in *T. pseudonana*, but which differs from the genome sequence in Thaps v3.0 at its 3′- termini. For this reason, we amplified full-length cDNA of nuPsb28 with the primer complement to adaptor on polyT primer. Genomic, EST and cDNA sequences were compared to find the probable mistake of sequence in EST library. To be sure that both versions of *psb28* are transcribed, we also amplified the plastid transcript from the cDNA (data not shown).

The nuclear-encoded Psb28 contains an extra 35 amino acids at its N-terminus, which clearly displays characteristics of bipartite targeting sequences to import proteins into diatom plastids. Diatom targeting sequences consist of a hydrophobic signal peptide domain (SP), followed by a transit peptide domain (TP). According to the SignalP 3.0 program [20] the nuPsb28 SP is composed of 18 amino acids, followed by a cleavage site between alanine (A) and phenylalanine (F) (Figure 1). These amino acids correspond to the SP cleavage site consensus based on experimental studies [21]. Furthermore, when the SP from nuPsb28 is compared with the proposed motif ASAF/AFAP from import experiments with *P. tricornutum* [21], [22], we see the conservation of a proline residue at the second position after phenylalanine. The putative TP sequence that follows is rich in the hydroxylated amino acids serine and threonine, which are known to be a common feature for chloroplast transit peptides in several organisms with primary or secondary plastids [23].

**Figure 4:**
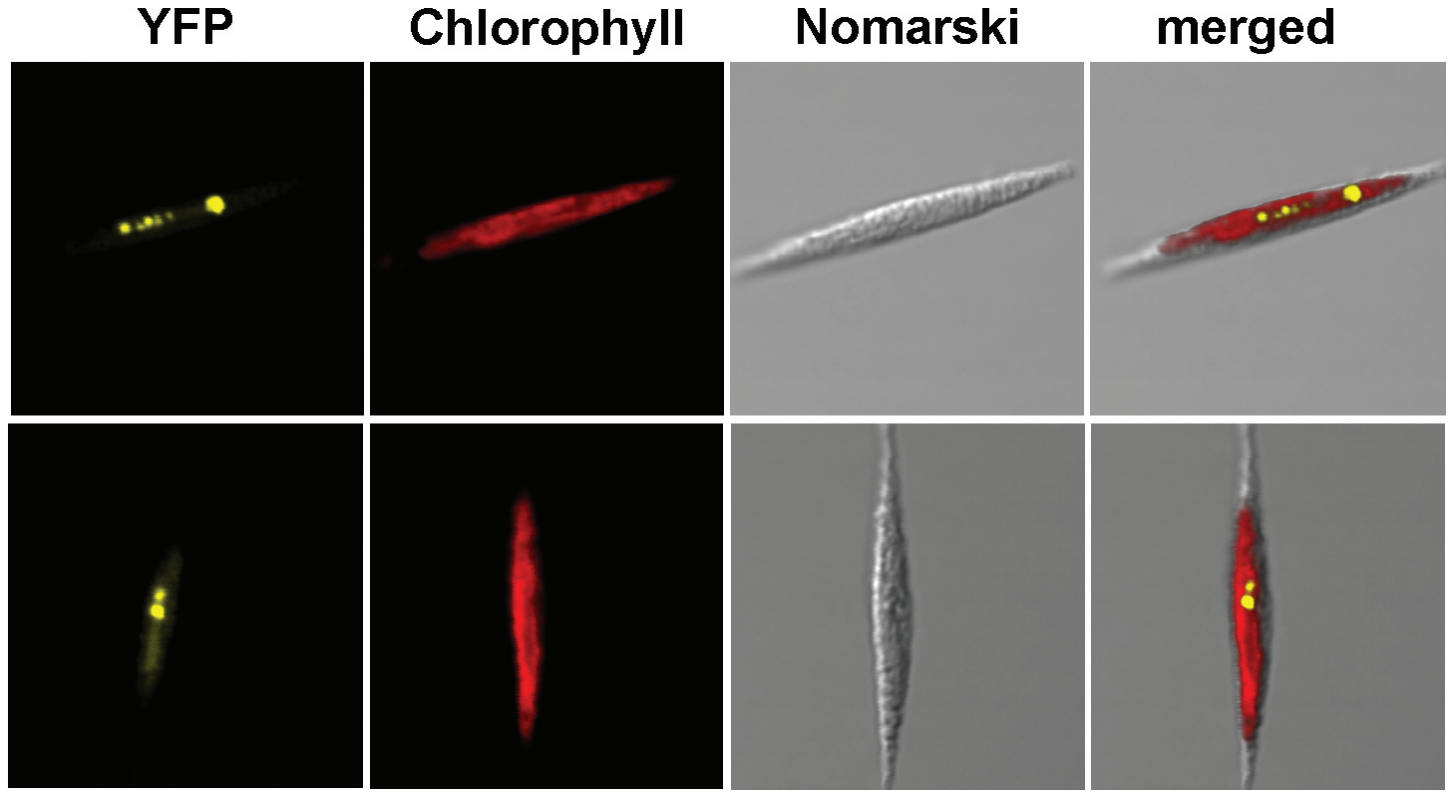
Subcellular localization of *T. pseudonana* nuPsb28 protein fused with EYFP in *P. tricornutum*. Merged pictures show co-localization of chlorophyll and EYFP fluorescence. It appears that the EYFP fusion protein accumulates principally inside the pyrenoid of the diatom plastid. Yellow: Yellow fluorescent protein fused with nuclear encoded Psb28 from *T. pseudonana*. Red: chlorophyll auto-fluorescence.

Experimental localization of nuPsb28 fused with yellow fluorescent protein (EYFP) at its C terminus showed that the protein is located inside the diatom complex plastid, in agreement with the *in silico* predictions (Figure 4). Although the observed EYFP signal is colocalized with chlorophyll autofluorescence, the signal is somewhat dispersed in the plastid stroma, and also accumulates in the pyrenoid.

## Discussion

It is believed that genes located in the plastid and other organelles tend to be transferred to the host nucleus, translated in the cytosol, and the proteins are posttranslationally targeted to the place of action, the organelle they originally came from. Evolutionary relics documenting such processes have been found following genome sequencing of various eukaryotes and their organelles [2]. We have investigated a rare case found in diatoms, where in *T. pseudonana* two *psb28* genes were found, one in the plastid genome, while the second appears to be localized in the nucleus and encodes a protein that is targeted to the diatom plastid [5]. Although it was suggested that these genes arose following a duplication event with one such duplicate being transferred to the nucleus by endosymbiotic gene transfer [5], we have detected substantial changes in the nucleotide composition between these genes – nuclear *psb28* is relatively GC rich while its homologue from the plastid genome is rich in ATs, resulting in a difference of 20% between these two genes. We speculate that these sequential changes have occurred thanks to different locations of the genes (plastid versus nucleus) or to make the protein targetable to the secondary diatom plastid. However, the recently documented presence of many genes derived from green algae in Chromalveolates open doors to alternative explanations [24]. It was suggested by the authors of this study that these green genes can represent remnants of an ancient endosymbiotic relationship between the ancestor of chromalveolates and a green alga [24]. It is supposed that such an endosymbiotic event preceded that with the red alga, which is dated to around 1 billion years ago. Thus the nuclear *psb28* gene could eventually originate from this green fraction. If this is the case, both versions of *psb28* would have coexisted in one cell for around 1 billion years, because such a green *psb28* would have to be present in a diatom genome already before the red endosymbiosis. We therefore suggest an origin of diatom nuclear *psb28* from ancient green endosymbiosis unlikely. The last but not least possibility is that nuclear *psb28* in diatoms originate via non-endosymbiotic horizontal gene transfer. Although such way of the gene acquisition can never be ruled out, there are several aspects of the *psb28*-based phylogenetic analyses suggesting low probablity of this event. First of all, there is only one robust placement of the diatom nuclear *psb28* sequences in our analyses: when the dataset was processed by Bayesian analysis using advanced models CAT and CATBP, all the diatom nuclear and plastid *psb28* sequences form short branches and cluster together, with posterior probabilities ranging from 0.77 and 0.98 for CAT and CATBP respectively (Figure 3). This was the only supported position of diatom nuclear *psb28* in our trees. The same but unsupported position (bootstrap 44%) was obtained by AsaturA program designed to deal with amino-acid saturation (Figure 3). This program constructed to uncover possible gene duplications allows to discriminate between mutationaly unsaturated positions and those residues that are likely to be saturated. Based on particular substitutional matrix and interactively modified substitution probability “cutoff” value, amino acid residues are sorted as “rare” or “frequent”. Thus the degree of saturation can be specified for different set of options and the original data can be divided into a saturated and unsaturated cluster. During a second step, the unsaturated data are used to compute pairwise distances and to construct conventional neighbor–joining tree [25]. All other phylogenetic analyses led to unsupported, and in our opinion, incorrect topologies: Maximum likelihood (ML) (figure 3A) and Maximum parsimony analyses (not shown) placed the nuclear *psb28* on a long branch in an unsupported sister position to the nuclear gene from *A. anophagefferens*, pelagophyte with red alga derived plastid, with the *psb28* sequence forming the longest branch in the tree (see Figure 3A for detailes). It is obvious that ML was not suitable to solve the phylogeny of nuclear *psb28* genes, while Bayesian analysis, when performed using appropriate models taking into account across-site heterogeneities in the process of amino-acid replacement, placed the diatom *psb28* to the position predicted by the concept of endosymbiotic gene transfer as it was originally expected. It appears that the selection of particular method and evolutionary model for the analysis is essential. Contrary to previously introduced substituional models based on an the assumption that all sites of the protein undergoes the same substitution process during evolution, the Bayesian mixture CAT model allows to describe distinct substitution processes at the amino-acid replacement occurring in different sites of a protein alignment [26]. In addition to that, CATBP model takes also heterogeneity among sequences (not sites only) into account [27], which makes it more suitable for the phylogenies that are affected by the compositional bias of the sequences and deals very well with LBA. Indeed, the usage of this model led to even better supported placement of the diatom nuclear *psb28* genes compared to CAT model (see Figure 3 for details). All in all, it appears that Bayesian inference with advanced CAT and CATBP models computed tree in agreement with the endosymbiotic gene transfer hypothesis, while all other methods used did not solved the phylogenetic position of diatom nuclear *psb28* genes at all.

It may not be overlooked that Psb28 is a very short protein containing 112 computable amino acid residues in the dataset matrix, from which only 82 characters are parsimony informative. The limited length of the analyzed gene consequently leads to lower or even no support of particular nodes in the tree, especially when the dataset contain high number of OTUs. Moreover, since the dataset contain also highly divergent sequences, phylogenetic information should be extracted using advanced evolutionary models in the frame of Bayesian analysis to deal with possible phylogenetic artefacts. Otherwise the artefactual signal can overlay the phylogenetic information, as it is in the case of ML and MP analyses.

**Figure 5:**
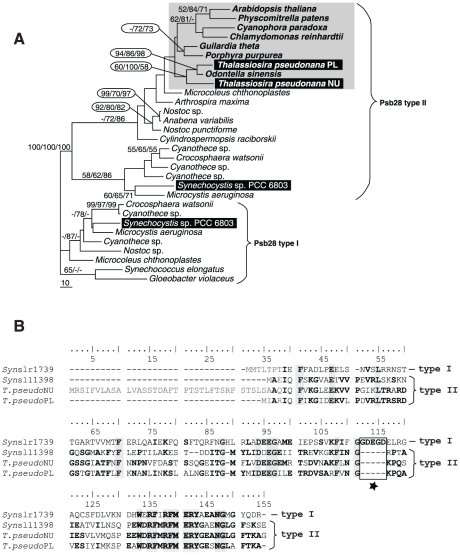
Two types of cyanobacterial Psb28 proteins. **A** Maximum parsimony phylogenetic tree based on cyanobacterial and eukaryotic Psb28 protein sequences (long branches excluded). The protein shows an occurrence of two basic sequential types (marked I and II). Numbers above branches indicate bootstrap supports as computed by Maximum parsimony (1000 replicates)/AsaturA (1000 replicates)/Maximum likelihood (500 replicates, WAG+I+Γ with all parameters estimated form the dataset). **B** The two types show low mutual similarity (identity of amino acids do not overcome 32%). The type I Psb28 contains specific insertions indicated by black star.

It should be noted that the presence of two *psb28* genes in one organism with its function restricted to a single organelle is extremely rare in photosynthetic eukaryotes and so far has been found only in two diatoms *T. pseudonana* and *F. cylindrus*. It is even possible that the two Psb28 proteins encoded in the diatoms could have acquired distinct functions and may retain in the genomes because of this reason. The nuPsb28 appears to be successfully targeted to the complex plastid where its own version is encoded and possibly expressed. Although both genes are transcribed and thus two distinct Psb28 can be functional in the plastid, the advantage of having another Psb28 encoded in the nucleus is not obvious. However, it has been shown that a *Synechocystis* sp. PCC 6803 contains two highly divergent Psb28 proteins displaying identity of only 33% with 48% of amino acid residues showing similar properties (positives) (see Figure 5 for details). Since there are two highly divergent proteins in *Synechocystis* sp. PCC 6803, distinct functions can be expected. Phylogenetic analysis of cyanobacterial *psb28* genes demonstrates the existence of two divergent types of Psb28 protein in these bacterial phototrophs (Figure 5A). It has been demonstrated that antibodies against Psb28 type I showed successful localization within a thylakoid membrane, while type II, which is homologous to the proteins found in diatoms and other eukaryotes, showed no signal [28]. It can suggests that only one gene (type I) is translated in *Synechocystis* sp. PCC 6803 or they are both differently expressed in reaction on various environmental conditions. Although the absence of Psb28 in *Synechocystis* sp. PCC 6803 double mutant did not affect the functional properties of PSII, the mutant showed accelerated turnover of the D1 protein, faster PSII repair, and a decrease in the cellular content of PSI. Recently, one of the two Psb28 proteins from *Synechocystis* sp. PCC 6803 was shown not to be a component of the fully assembled photosytem II complex, but rather to be preferentially bound to the assembly intermediate inner antenna CP47 [28]. Thus the possibilty of different functions of the two Psb28 proteins in diatoms is still in game and cannot be rejected with certainty. However, since both diatom proteins belong to the same type II, and such arrangement was found only in the two diatoms and was never referred for any other eukaryotic phototroph including red algae, the supposed predescedor of a diatom complex plastid, we postulate that this phenomenon represents an intermediate state reflecting the ongoing endosymbiotic gene transfer and that the plastid-encoded version will eventually be lost from the genome.

Both versions of the *psb28* gene have been found in the centric diatom *T. pseudonana* and pennate diatom *F. cylindrus*, but the nuclear *psb28* gene is absent from the pennate *P. tricornutum* suggesting that this gene has been either lost from *P. tricornutum* during evolution, or the plastid *psb28* has been transferred to the diatom nucleus several times independently. Since the data from other diatoms are not available, we cannot decide between these two scenarios with certainty. However, it was shown in angiosperms that short genes such as infA (approximately 100 amino acids) can be concurrently lost from the plastid genomes with consequent multiple independent transfers to the host nucleus [29]. We expect that the process of endosymbiotic gene transfer continues in diatoms and we therefore prefer independent transfer of *psb28* to the diatom nucleus in various lineages.

Comparison of diatom plastid genomes with other heterokont lineages has led to the detection of many gene losses and rearrangements [12]. The endosymbiotic transfer of the *psb28* gene into the nucleus might be one of the more distinct examples. It has occurred once in the green lineage of Archaeplastida, after the glaucophytes and rhodophytes diverged. In contrast, rhodophytes retained *psb28* in their plastid genome at the time of the secondary endosymbiotic event. However, the transfer of *psb28* from the plastid to the nuclear genome has already happened in two heterokonts: the haptophyte *E. huxleyi* and the pelagophyte *A. anophageferrens*. When compared to the diatom plastid genomes, *E. huxleyi* contains fewer genes in the plastid. Seemingly therefore, the process of endosymbiotic gene transfer is more advanced here [12].

The mechanism of transfer of a plastid gene into the nucleus has been already described in tobacco [30]. Plastid DNA integration into the nuclear genome appears to be similar to what occurs following biolistic transformation of plant cells with exogenous DNA, but no sequence preference was identified near junctions [30]. Surprisingly, there is evidence for newly transposed fragments of plastid DNA in male gametes being more frequent than in female gametes of tobacco [31]. This could be a prevention of their escape through unequal inheritance of endosymbiotic organelles during gametogenesis. It is known that sperm and pollen possess less cytoplasm than eggs. Similarly, centric diatoms such as *T. pseudonana* create flagellated microgametes resembling sperm as well as larger egg-like macrogametes, whereas pennate diatoms form non-flagellated morphologically identical gametes known as isogametes [32]. Contribution of the cytoplasmic material should therefore be equal. This may be the difference between the major groups of diatoms in probable transitions in germ lines. Experimentally transferred DNA in tobacco was also shown to be unstable and only retained for a few lineages [31]. However, the driving force of transitions and rearrangements in diatom genomes appear to differ from that in higher plants, as there is significant divergence between the pennate and centric species, which can also contribute to the level of gene transfer [14].

We conclude that *psb28* from *T. pseudonana* is a gene in the process of endosymbiotic gene transfer. This gene is present in both nuclear and plastid genomes of *T. pseudonana* and both are transcribed, with the nuclear-encoded protein being targeted to the complex plastid. We can speculate that it is the fusion of Psb28 with the ribosomal protein S4 that prevents the elimination of *psb28* from the plastid genome. Phylogenetic analyses show that nuclear *psb28* is a duplicate of the plastid homologue, although this relationship only became apparent when appropriate methods were used. The reason lies in the compositional bias of the analyzed sequences, which causes LBA and substantially affects the topology of the tree.

## Materials and Methods

### Molecular Phylogeny

Nucleotide sequences coding for Psb28 from various photoautotrophs including cyanobacteria, glaucophytes, plants, chlorophytes, rhodophytes, cryptophytes, haptophytes, and stramenopiles, were downloaded from GenBank™ and other sources (see Table S1). The nucleotide sequence dataset was translated to amino acids in BioEdit [33] and either aligned using ClustalW algorithm (BioEdit) and retranslated back into nucleotides or aligned using Mafft-6.717 [34]. Both amino acid and nucleotide alignments were used for further phylogenetic analyses. Both alignments were manually edited (BioEdit), and ambiguously aligned regions and gaps were excluded from further analyses. A specific nucleotide dataset was made by exclusion of the third nucleotide codon positions. Nucleotide datasets were used to construct Maximum parsimony (MP) [35] and Maximum likelihood trees (ML with GTR+Γ^4^+I model) [36]. Because the third codon position appeared to be saturated, we excluded this position prior to the analysis (trees not shown). Amino acid alignments were used to construct MP [35], Neighbor joining (AsaturA [25]; method designed to deal with saturation of amino acids with LG model [37]); Bayesian (CAT-BP model as implemented in NH PhyloBayes 0.2.1 [27]; CAT model in PhyloBayes 3.2d [26]) and ML trees using PhyML program [36] with WAG model and discrete gamma distribution in 4 categories; all parameters were estimated from the dataset. Convergence of the chains in the Bayesian analyses was assessed by monitoring of both the topology and posterior probabilities during the analyses and by comparing two independent chains using ‘bpcomp’ (PhyloBayes 3.2d) or ‘compchain’ (NH Phylobayes 0.2.1). To explore the evolution of Psb28 in the context of cyanobacteria we have constructed dataset comprising various cyanobacteria and selected eukaryotes. The sequences downloaded from the GeneBankTM (see Table S2 for details) were aligned using Mafft-6.717 [34], the alignment was manually edited [33] and used to compute Maximum parsimony [35], Maximum likelihood [36] and AsaturA trees [25].

### Cultures and media

*Thalassiosira pseudonana* Hasle et Heimdal CCMP1335 and *Phaeodactylum tricornutum* Bohlin CCMP632 were provided by Provasoli-Guillard National center for Culture of Marine Phytoplankton (ME, USA). Both axenic cultures were grown in plastic 150 ml flasks filled with artificial sea water medium, made by dissolving “Tropic marine” salt (Wartenberg, Germany) at 35 units of practical salinity. Additionally, medium was enriched by Guillard's (F/2) Marine Water Enrichment Solution (Sigma-Aldrich). Cultures were grown under standard conditions at 18°C with cool white fluorescent light (120 µmol m^−2^ s^−1^), and a 12 h light/12 h dark photoperiod.

### RNA isolation and reverse transcription

For RNA isolation cells were harvested by centrifugation at room temperature for 10 min at 5,000 rpm. Total RNA from *T. pseudonana* was isolated by Tri Reagent (Molecular Research Center Inc.) according to the manufacturer's instructions. A total of 10^7^ cells was used as starting material for 1 ml of Tri Reagent. The final concentration of RNA was measured by spectrophotometry. The polyT primer with adaptor, nucleotides, total RNA and reverse transcriptase Superscript II (Invitrogen) were used for cDNA synthesis. After cDNA synthesis the product was treated with RNase H for 10 min at 37°C.

### PCR and pENTRY cloning

The nuclear *psb28* gene was amplified from the *T. pseudonana* cDNA using primers PSB1 CACCATGAGATCAATCTTCGTCCTCG and PSB2 AGCCTTGGTGAACCCAAGTCCATT. To clone the PCR product into pENTR vector (Invitrogen), primers were designed according to the manufacturer's instructions, and in order from the first nucleotide of the start codon to the triplet of the last codon. The presence and orientation of the nuclear *psb28* gene in the pENTR vector was confirmned by sequencing (data not shown). Thereafter pENTR with nuclear *psb28* was recombined with Destination vector pDEST- CEYFP [38] using the LR recombination reaction (Invitrogen) to produce the vector overexpressing the nuclear-encoded Psb28 protein (nuPsb28) fused to enhanced yellow fluorescent protein (EYFP) at the C terminus. The resulting LR product was propagated and purified from chemically competent *E. coli* strain TOP10. In addition, we used the PSB1 and primer 5′- GCGAGCACAGAATTAATACGACT-3′, which is complementary to the adaptor sequence to amplify the expressed product of nuclear *psb28*. The PCR product was cloned into pGem-Easy vector (Promega) and thereafter sequenced.

### Diatom co-transformation

Nuclear co-transformation of *P. tricornutum* was performed with expression vector nuPsb28-C-EYFP and resistance vector pFCPFp-Sh ble, as previously described [18]. The vector mix was introduced by microparticle bombardment using a Biolistic PDS-1000/He Particle Delivery System (Bio-Rad, Hercules, CA, USA). For selection of positive transformants, bombarded cells were plated onto ½ artificial seawater (ASW) agar plates (1% agar) supplemented with 100 µg/mL phleomycin (Duchefa). After 2–3 weeks of incubation in standard growing conditions (see cultures and media for details), resistant clones formed colonies, which were subsequently inoculated into liquid ASW medium and examined by confocal microscopy.

### Microscopy

Cellular localization of nuPsb28-C- EYFP fusion proteins were analyzed with a confocal system FluoView™ FV1000 configured with an inverted mobile IX81 microscope (Olympus). A scanning laser with wavelength 515 nm was used for excitation of chlorophyll and EYFP. The emitted fluorescence was detected using a bandwidth setting of 525–571 nm for EYFP, and 620–710 nm for chlorophyll autofluorescence. Images were generated by the Olympus FV10-ASW Version 01.07.01.00 software and subsequently processed. The final picture arrangement was made using Adobe Photoshop CS2.

## Supporting Information

Table S1 Sequences used for phylogenetic analysis of Psb28.
(0.06 MB DOC)

Table S2 Sequences used for phylogenetic analysis of cyanobacterial Psb28 proteins.
(0.05 MB DOC)
