# Supplementary material for: A Gene in the Process of Endosymbiotic Transfer
Source: PLoS One. 2010 Oct 6;5(10):e13234. doi: 10.1371/journal.pone.0013234 (PMC2950852; doi:10.1371/journal.pone.0013234)
Supplement: Table S1 — Sequences used for phylogenetic analysis of Psb28. (0.06 MB DOC) [file pone.0013234.s001.doc]

Table S1. Sequences used for phylogenetic analysis of Psb28.

| OTU | classification | accession |
| --- | --- | --- |
| *Anabaena variabilis* | Cyanobacteria | NC_007413 |
| *Arabidopsis thaliana* | Spermatophyta | AL161573 |
| *Aureococcus anophagefferens* | Stramenopila | jgi|Auran1|22066 |
| *Chlamydomonas reinhardtii* | Chlorophyta | XM_001690485 |
| *Chlorella* sp. | Chlorophyta | jgi|ChlNC64A_1|140795|  IGS.gm_3_00534 |
| *Coccomyxa* sp. | Chlorophyta | jgi|712_1_CBOZ_CBPA |
| *Cyanidium caldarium* | Rhodophyta | NC_001840 |
| *Cyanidioschyzon merolae* | Rhodophyta | NC_004799 |
| *Crocosphaera watsonii* | Cyanobacteria | NZ_AADV02000138 |
| *Cyanophora paradoxa* | Glaucophyta | NC_001675 |
| *Ectocarpus siliculosus* | Stramenopila | FP102343 |
| *Emiliania huxleyi* | Haptophyta | jgi|Emihu1|48859|gw1.4.121.1 |
| *Fragilariopsis cylindrus* (pl) | Stramenopila | jgi| scaffold_95 |
| *Fragilariopsis cylindrus* (nu) | Stramenopila | jgi|scaffold_108|1|91266 |
| *Fucus vesiculosus* | Stramenopila | FM957154 |
| *Galdieria sulphuraria* | Rhodophyta | MSU_GD_stig_35 |
| *Gracilaria tenuistipitata* | Rhodophyta | NC_006137 |
| *Guilardia theta* | Cryptophyta | NC_000926 |
| *Heterosigma akashiwo* | Stramenopila | NC_010772 |
| *Micromonas pusilla* | Chlorophyta | jgi|MicpuC2|19538|e_gw1.9.524.1 |
| *Micromonas* sp. | Chlorophyta | XM_002503059 |
| *Odontella sinensis* | Stramenopila | NC_001713 |
| *Osterococcus lucimarinus* | Chlorophyta | XM_001421666 |
| *Phaeodactylum tricornutum* | Stramenopila | NC_008588 |
| *Physcomitrella patens* | Bryophyta | XM_001765887 |
| *Populus trichocarpa* | Spermatophyta | XM_002303073 |
| *Porphyra purpurea* | Rhodophyta | NC_000925 |
| *Porphyra yezoensis* | Rhodophyta | NC_007932 |
| *Rhodomonas salina* | Cryptophyta | NC_009573 |
| *Thalassiosira pseudonana* (pl) | Stramenopila | NC_008589 |
| *Thalassiosira pseudonana* (nu) | Stramenopila | XM_002290686 |
| *Vaucheria litorea* | Stramenopila | EU912438 |
| *Vitis vinifera* | Spermatophyta | XM_002271630 |
| *Volvox carteri* | Chlorophyta | jgi|Volca1|103002|  estExt_fgenesh4_pg.C_30157 |
| *Zea mays* | Spermatophyta | EU955345 |
