# Supplementary material for: A Gene in the Process of Endosymbiotic Transfer
Source: PLoS One. 2010 Oct 6;5(10):e13234. doi: 10.1371/journal.pone.0013234 (PMC2950852; doi:10.1371/journal.pone.0013234)
Supplement: Table S2 — Sequences used for phylogenetic analysis of cyanobacterial Psb28 proteins. (0.05 MB DOC) [file pone.0013234.s002.doc]

Table S2. Sequences used for phylogenetic analysis of cyanobacterial Psb28 proteins.

| OTU | classification | accession |
| --- | --- | --- |
| *Anabena variabilis* | Cyanobacteria | YP_321112 |
| *Arthrospira maxima* | Cyanobacteria | ZP_03274793 |
| *Crocosphaera watsonii* | Cyanobacteria | EAM48515 |
| *Crocosphaera watsonii* | Cyanobacteria | ZP_00516740 |
| *Cylindrospermopsis raciborskii* | Cyanobacteria | EFA70375 |
| *Cyanothece* sp. | Cyanobacteria | ACB50949 |
| *Cyanothece* sp. | Cyanobacteria | ACV00163 |
| *Cyanothece* sp. | Cyanobacteria | ACK71343 |
| *Cyanothece* sp. | Cyanobacteria | YP_002379748 |
| *Cyanothece* sp. | Cyanobacteria | ZP_01730583 |
| *Gloeobacter violaceus* | Cyanobacteria | NP_923987 |
| *Microcoleus chtonoplastes* | Cyanobacteria | EDX71357 |
| *Microcystis aeruginosa* | Cyanobacteria | CAO90585 |
| *Microcystis aeruginosa* | Cyanobacteria | CAO86551 |
| *Nostoc* sp. | Cyanobacteria | NP_485125 |
| *Nostoc* sp. | Cyanobacteria | Q8YYP5 |
| *Nostoc punctiforme* | Cyanobacteria | B2J567 |
| *Synechocystis* sp. PCC 6803 | Cyanobacteria | (slr1739) NP_441099 |
| *Synechocystis* sp. PCC 6803 | Cyanobacteria | (sll1398) NP_439938 |
| *Synechococcus* sp. | Cyanobacteria | YP_172336 |
| photosynthetic eukaryotes | | |
| *Arabidopsis thaliana* | Spermatophyta | AL161573 |
| *Chlamydomonas reinhardtii* | Chlorophyta | XM_001690485 |
| *Cyanophora paradoxa* | Glaucophyta | NC_001675 |
| *Guilardia theta* | Cryptophyta | NC_000926 |
| *Odontella sinensis* | Stramenopila | NC_001713 |
| *Physcomitrella patens* | Bryophyta | XM_001765887 |
| *Porphyra purpurea* | Rhodophyta | NC_000925 |
| *Thalassiosira pseudonana* (pl) | Stramenopila | NC_008589 |
| *Thalassiosira pseudonana* (nu) | Stramenopila | XM_002290686 |
